# X-ray lasers and crystallography

**DOI:** 10.1107/S2052252514009567

**Published:** 2014-04-30

**Authors:** John C.H. Spence

**Affiliations:** a Department of Physics, Arizona State University, Tempe, AZ 85282, USA

**Keywords:** X-ray lasers, XFELs, crystallography

## Abstract

The development of X-ray lasers and their applications in crystallography is described. In the birth of this new field, **IUCrJ** is ideally positioned to present this research to both specialists in crystallography, and to the wider audience in structural biology.

The invention of the laser in the 1950 s for visible light and microwaves, and the slow but steady recognition of its manifold uses, is a truly remarkable story in the history of science. But the severe λ^3^ dependence of the ratio of stimulated (mostly coherent) to spontaneous (incoherent) emission meant that efforts to build an X-ray laser seemed hopeless for decades. As so often happens in the history of science, the breakthrough eventually occurred at the interface of several fields – synchrotron science (and especially their insertion devices), laser physics, and work on microwave tubes for radar, emerging from the second world war. Synchrotrons themselves were an outgrowth of the particle accelerators of nuclear physics, whose X-ray radiation was considered a nuisance. All of this culminated recently in the construction of the first hard-X-ray laser, the US Department of Energy’s Linac Coherent Light Source (LCLS), at their SLAC laboratory near Stanford. The first X-ray lasing occurred in that two-mile long tunnel on April 21, 2009, at about 2 kV, in an all-or-nothing moment of intense excitement, as theoretical predictions proved spot-on. The new laser principle needed for hard-X-ray lasing, the free-electron laser (FEL), was first demonstrated in the infra-red region at Stanford in 1975 in John Madey’s group, following earlier theoretical work by Motz and Phillips on microwave tubes. Other FELs soon followed, in the microwave and visible region, leading to the LCLS. The XFEL method provides brief pulses of X-ray laser radiation by the SASE (self-amplified spontaneous emission) process, using a resonant undulator driven by a LINAC electron accelerator. Each LCLS pulse, of 10 fs duration (repeated 120 times a second) contains about 10^12^ hard-X-ray photons, about the same number that a synchrotron might generate in a second.

The gamble taken by the DOE in committing to the $600 × 10^6^ construction of the LCLS around 2004 was laudable, in these days when low-risk incremental science seems the only way to attract funding against high odds. Will it lase? Will it be useful? The past five years have seen their vision vindicated with breakthough applications in many fields, from materials science and atomic and molecular physics to condensed matter physics and biology. Similar machines are now under construction around the world, or are already operating (in Japan), including those starting soon at DESY in Hamburg (EXFEL), in Switzerland (SwissFEL), and now a second machine at SLAC (LCLS 2).

The first applications of interest to crystallographers soon appeared in publications in 2011. These were proof-of-principle applications to the goal of getting one snapshot diffraction pattern from a single virus, and to hydrated membrane protein nanocrystals, some as small as a dozen unit cells on a side. But the remarkable discovery was soon made that this use of snapshots (from a micron or submicron diameter beam) provided the opportunity to outrun radiation damage, even at high resolution. A useful diffraction pattern could be collected during the femtosecond pulse, before the onset of the most important radiation damage processes. Following earlier theoretical predictions, this effect had been demonstrated experimentally in 2006 at low resolution using V-UV SASE radiation at the DESY FEL (FLASH), and many papers have since elucidated the time-evolution of the damage processes at the atomic, molecular and bulk scales. The exciting implication was that crystallographers could use snapshots instead of freezing to avoid damage, which, along with crystal quality, have always limited the resolution and quality of crystallographic data. This in turn opens the way to the study at room temperature of structural dynamics with very high time resolution, without significant radiation damage, for molecules in their natural environment. At the same time, the structural biology community has responded to the opportunity offered by the ability to study nanocrystals with the development of new methods for growing these ‘invisible’ sub-micron crystals.

But this new ‘diffract-then-destroy’ mode of doing crystallography has created severe challenges for sample delivery, since every sample is immediately vaporized by the beam after producing its diffraction pattern. (The focused XFEL beam alone drills holes in sheet steel with every shot). A rich variety of experimental schemes have been developed to deal with this need to deliver hundreds of samples (or far more, at the new superconducting high-repetition rate facilities) every second. These include rapidly scanned goniometer stages, and liquid, gas or lipid-cubic phase continuous jets, of micron-diameter, which spray sample in vacuum across the pulsed X-ray beam. Three main modes of operation have evolved so far – fast solution scattering (FSS), serial femtosecond diffraction from nanocrystals (SFX), and snapshots with one bioparticle, such as a virus, per shot (single particle or SP mode). For each mode, a time-resolved variant is possible, for which early pump-probe SFX results have now been published. Finally, the work has introduced new challenges in diffraction physics. The scattering from nanocrystals, for example, consists of thousands of patterns (each from a different nanocrystal of different size, randomly oriented) showing only partial reflections, which must be merged in three-dimensions. A ‘Monte Carlo’ approach, suggested in 2010, has mainly therefore been adopted to average out the fluctuations in the many stochastic experimental parameters which XFEL data presents us with (including large shot-to-shot intensity variations), and efforts to improve on this continue. The smallest nanocrystals show clear ‘shape-transform’ effects, whose fringes, running between Bragg reflections, provide new opportunities for solving the phase problem. For single particle patterns, the challenge of determining the orientational relationship between successive shots has been solved by ingeneous advanced theoretical methods provided by several groups.

Most of this work over the last decade of development has been in biophysics, and only very recently have the major funding agencies in structural biology started to take an interest. But a few examples of ‘new biology’ are now starting to appear from these exciting new methods, many in this journal – the solution of new GPCR structures from human protein which fail to grow crystals large enough for conventional crystallography, the observation of glycoselation and new structures at 1.9 Å resolution in an enzyme drug target for sleeping sickness (using *in-vivo* crystallization, facilitated by liquid jet sample delivery), the solved structure of a new opiod receptor for new analgesics, and time-resolved high-resolution imaging of the processes of photosynthesis by both the FSS and SFX methods. Data from two-dimensional protein monolayers has been collected, with exciting possibilities for synchronized dynamic imaging. Equally important, the time required for data collection has been reduced over the past four years from several days to a few hours, often with atomic resolution. Using the kilohertz readout cameras of the new XFELs which start operation around 2016, we expect this time to be reduced to a few minutes. The first of a series of annual conferences on this new ‘BioXFEL’ field was held in October 2013 at the Royal Society in London. As always, the birth of a new field is the most exciting time to be involved, and our new journal **IUCrJ** is ideally positioned to continue its leadership in presenting these papers to both specialists in crystallography, and to the wider audience in structural biology.

